# The Improvement of Nanoemulsion Stability and Antioxidation via Protein-Chlorogenic Acid-Dextran Conjugates as Emulsifiers

**DOI:** 10.3390/nano10061094

**Published:** 2020-06-01

**Authors:** Chang Liu, Hua Jin, Yue Yu, Jingying Sun, Huanyu Zheng, Yan Zhang, Jing Xu, Xiuqing Zhu

**Affiliations:** 1 College of Art and Science, Northeast Agricultural University, Harbin 150030, Heilongjiang, China; lovekursaal@163.com (C.L.); jinhua@neau.edu.cn (H.J.); Y13206689606@163.com (Y.Y.); asunjinying@126.com (J.S.); 2 College of Food Science, Northeast Agricultural University, Harbin 150030, Heilongjiang, China; zhenghuanyu1@163.com; 3 Heilongjiang Green Food Science Research Institute, Harbin 150028, Heilongjiang, China; 4 National Research Center of Soybean Engineering and Technology, Harbin 150028, Heilongjiang, China; 5 Coastal Research and Extension Center, Mississippi State University, Starkville, MS 39762, USA; yzhang@fsnhp.msstate.edu; 6 Key Laboratory of Grain Food and Comprehensive Processing of Grain Resource of Heilongjiang Province, College of Food Engineering, Harbin University of Commerce, Harbin 150076, Heilongjiang, China

**Keywords:** protein, chlorogenic acid, dextran, nanoemulsion, stability, antioxidation

## Abstract

In this experiment, the peanut protein isolate (PPI), soybean protein isolate (SPI), rice bran protein isolate (RBPI), and whey protein isolate (WPI) were modified by linking chlorogenic acid covalently and linking dextran by Maillard reaction to prepare protein-chlorogenic acid-dextran (PCD) conjugates. As for structures, conformational changes of conjugates were determined by Sodium Dodecyl Sulfate-Polyacrylamide Gel Electrophoresis (SDS-PAGE), Fourier transform infrared (FT-IR), and fluorescence measurements. The molecular weights of PCD conjugates became larger, the structure became disorder, and the amino acid residues inside the protein were exposed to the polar environment when compared to protein-chlorogenic acid (PC) and native proteins (NPs). As for properties, the interfacial tension reduced and antioxidant activity of PCD conjugates enhanced in varying degrees. Based on this, PCD conjugates were used as emulsifiers in order to investigate the properties of nanoemulsions and compared with PC conjugates and NPs. The mean droplet diameters (MDD) results showed that the nanoemulsions that were stabilized by PCD conjugates had the smallest particle sizes and exhibited uniformly dispersed spherical shapes. The storage and oxidative stabilities of PCD conjugates were also significantly improved. In comparison, nanoemulsion that was stabilized by PPI-chlorogenic acid-dextran conjugate had the smallest particle size and optimal stability among four protein stabilized nanoemulsions.

## 1. Introduction

Nowadays, the nanoemulsion-delivery system has received widespread attention on encapsulating, protecting, and releasing lipophilic active ingredients, which was mainly due to their high stability and superior protective effects on carrier nutrients when compared to conventional emulsions [1]. Therefore, the nanoemulsion-delivery system has great potential to act as carriers for various drugs and nutrients, and then rapidly developed during the past few decades. The selection of emulsifiers in nanoemulsions is always a hot topic for nanoemulsion research [2]. Increasing amounts of researchers tended to focus on non-toxic, readily available, and food-grade emulsifiers of nanoemulsions [3,4]. Subsequently, many amphiphilic proteins are used for nanoemulsion fabrication that is based on their excellent adsorption on the oil droplet surface to prevent aggregation [5].

Some certain processing and storage conditions could alter the protein molecular conformation, aggregation state, and functional properties [6]. The widespread use of protein as emulsifier was then limited. Therefore, there has been great interest in improving the functional properties of proteins by modification, such as conjugating with polyphenols or polysaccharides. Polyphenols were considered to inhibit oxidation and protect the interfacial structure of the emulsion. For instance, Li et al. [7] found that non-covalent binding of curcumin and β-lactoglobulin showed significant improvement of the 2,2′-amino-di(2-ethyl-benzothiazoline sulphonic acid-6)ammonium salt (ABTS) antioxidant activity for the conjugates. Fan et al. [8] found that the non-covalent combination of whey protein and epigallocatechin gallate (EGCG) could effectively improve the oxidative stability of the emulsions. However, previous studies have also shown that the reversibility of non-covalent binding between proteins and polyphenols could lead to the instability of the conjugates at food processing conditions [9]. In this case, covalent attachment of the protein to polyphenols might be considered as a better method. Recently, research had shown that the covalent attachment of the protein to polyphenols showed greater potential in inhibiting lipid oxidation [10], which could improve the emulsion antioxidant properties and stability [11]. Ahmed et al. [9] prepared covalently β-lactoglobulin-phenol conjugates, which showed better emulsification, oxidation resistance, and thermal stability than non-covalent conjugates.

The protein-polysaccharide conjugates that were produced by Maillard reaction have attracted attention due to their increased functional properties, such as antioxidant activity, emulsifying properties, and thermal stability [12,13]. The protein-polysaccharide conjugates effectively promoted the protein more firmly to adsorb on the oil-water interface, which could improve the stability of the emulsion and prevent droplet aggregation owing to the steric repulsion [13]. Yang et al. [14] obtained soy protein isolate-soy soluble polysaccharide conjugates by Maillard reaction and prepared the emulsion with higher physical stability.

In summary, the addition of polyphenols and polysaccharides both have effects on the protein structure and they could improve the protein emulsifying and other functional properties. Therefore, it was necessary to investigate the influence of protein-polyphenol-polysaccharide conjugates on the capability of emulsions preparation. Presently, it had been studied that lactoferrin-chlorogenic-dextran conjugate could improve the stability and bioaccessibility of β-carotene nanoemulsions as emulsifiers [15]. However, such studies on plant proteins have not been reported. Especially, the effect of conjugates structure and functional properties is not systematically evaluated for emulsion stability and properties. Thus, in our experiment, the protein-chlorogenic acid-dextran conjugates were formed, and the structures and properties of the modified proteins were then characterized. Finally, the physicochemical stability of the nanoemulsions stabilized by modified proteins was studied to analyze the influence of emulsifier structure and properties on emulsion.

## 2. Materials and Methods

### 2.1. Materials

Defatted peanut powder and rice bran powder were purchased from Changshou Food Co. (Qingdao, China). Low temperature defatted soybean powder was purchased from Harbin High-Tech Co. (Harbin, China). Whey protein isolate was purchased from Jiangda Biotechnology Co. (Zhengzhou, China). Corn oil was purchased from a local grocery. Chlorogenic acid was purchased from Solarbio Co. (Beijing, China). Dextran powder was purchased from Sinopharm Chemical Reagent Co. (Shanghai, China). 1,1-Diphenyl-2-picrylhydrazyl radical 2,2-Diphenyl-1-(2,4,6-trinitrophenyl)hydrazyl (DPPH), and 2,2′-amino-di(2-ethyl-benzothiazoline sulphonic acid-6)ammonium salt (ABTS) solid powders were purchased from Biotopped Co. (Beijing, China). Nile red/blue were purchased from Sigma–Aldrich (St. Louis, MO, USA). Butylated hydroxytoluene (BHT) was purchased from Merck Co. (Darmstadt, Germany). Trichloroacetic acid (TCA) was from Samchun Pure Chemicals Co. (Seoul, Korea). Analytical-grade reagents were used, unless otherwise stated.

### 2.2. Preparation of Conjugates

#### 2.2.1. Native Proteins (NP)

The isolated proteins of peanut (PPI), soybean (SPI), and rice bran (RBPI) were extracted based on previous research [16,17,18]. Isolated proteins were prepared by the alkali dissolution-acid precipitation method. In brief, added water to defatted peanut powder, soybean powder, and rice bran powder, and then adjusted the pH to 9.5, 8.0, and 9.0 with 2.0 M NaOH, respectively. Stirred and centrifugated at 4000 rpm for 15 min. to obtain the protein supernatant. Subsequently, the pH of solutions was adjusted to 4.5 with 2.0 M HCl and centrifugated at 4000 rpm for 20 min. to obtain the protein precipitate. Added distilled water to the precipitation and adjusted to pH 7.0 with 2.0M NaOH to ensure it is completely dissolved, and then lyophilized to obtain the corresponding protein isolate.

#### 2.2.2. Protein-Chlorogenic Acid Conjugates (PC)

The PC conjugates were prepared according to the method of Liu et al. [15] with slight modifications. 1.0 g of protein (PPI, SPI, RBPI, WPI) was dissolved in 50 mL of distilled water, respectively, stirred at room temperature for 4 h, and then adjusted to pH 9.0 with 0.1 M NaOH. Meanwhile, 0.25 g chlorogenic acid (CA) solution was dissolved in 50 mL of distilled water, adjusted pH to 9.0 with 0.1 M NaOH. The protein solution was then mixed with the CA solution, continuously stirred at room temperature for 24 h, and then dialyzed against distilled water for 48 h to remove free CA. After that, the samples were stored in the refrigerator at −80 °C for pre-freezing, and then lyophilized by Telstar’s LYOQUEST-85PLUS Lyophilizer at −80 °C for 24 h to obtain the PC samples.

#### 2.2.3. Protein-Chlorogenic Acid-Dextran Conjugate (PCD)

The PCD conjugates were prepared according to the method of Liu et al. [15], with slight modifications. The Protein–CA conjugate and the dextran powder (Dex, Mw = 40 kDa) were respectively dissolved in distilled water at a concentration of 20 mg/mL and stirred at room temperature for 4 h. Subsequently, the PC conjugate solution was mixed with the dextran solution at a ratio of 1:2 (*v*/*v*). The solution was adjusted to pH 7.0 and lyophilized. The obtained powder was incubated for 24 h at 60 °C, and the required 79% relative humidity (RH) was provided by saturated KBr solution.

### 2.3. The Conjugates Structural Analysis

#### 2.3.1. Sodium Dodecyl Sulfate-Polyacrylamide Gel Electrophoresis (SDS-PAGE)

SDS-PAGE gel electrophoresis analysis was performed according to the Laemmli method [19]. The NP, PC, and PCD samples solutions (5 mg/mL) was mixed with sample buffer in equal proportions, boiled for 5 min., and then cooled to room temperature. 10 μL of the sample was taken and run at 80 V and 120 V constant voltages of stacking gel and separation gel, respectively. The strips were stained with Coomassie Brilliant Blue R-250 and scanned with a Gel Doc EZ Imager (BIO-RAD, Shanghai, China).

#### 2.3.2. Fourier Transform Infrared (FT-IR) Spectroscopy Measurement

The NP, PC, and PCD freeze-dried samples were analyzed by Fourier transform infrared (FT-IR) spectroscopy using a Bruker Vertex 70 FT-IR spectrometer (Bruker Optics, Ettlingen, Germany). The FT-IR spectra were recorded with 64 scans at 4 cm^−1^ resolution from 4000 cm^−1^ to 400 cm^−1^ at 25 °C. Peakfit Version 4.12 (Systat Software Inc., California, USA, 2007) was used to perform baseline correction, deconvolution, and second derivative on the spectrum of the amide I band (1700–1600 cm^−1^). Gaussian peak shape was used to fit the number and position of sub-peaks and calculate the peak area. The corresponding relationship between each sub-peak and secondary structure were: 1610–1638 cm^−1^ and 1680–1690 cm^−1^ corresponding to β-sheet; 1640–1648 cm^−1^ corresponded to random curl; 1650–1660 cm^−1^ corresponded to α- helix; and, 1660–1680 cm^−1^ and 1690–1700 cm^−1^ corresponded to β-turns [20].

#### 2.3.3. Fluorescence Measurements

The fluorescence spectra of the NP, PC, and PCD samples were determined using a F-4500 Fluor photometer (Hitachi, Tokyo, Japan) according to Bonomi’s research with modifications [21]. The samples (0.2 mg/mL) were dissolved in 10 mM phosphate buffer (pH 7.0) according to the method proposed by Zhou et al. [22]. The sample solutions were scanned at an excitation wavelength of 290 nm and the emission spectra were recorded from 300 to 500 nm at a constant slit of 5 nm for both excitation and emission.

### 2.4. Interfacial Tensions (IT) Measurement

The interfacial tension of the NP, PC, and PCD samples were determined using a Du Nouy ring tensiometer (TP681, Timepower, Co., Let., Beijing, China) at 25 °C. The loop was cleaned with acetone and then dried in a blue flame before each measurement. Repeat the experiment until the constant reading was obtained.

### 2.5. The Activities of Antioxidant

#### 2.5.1. DPPH Radical Scavenging Activities

1 mL of the NP, PC, and PCD samples were taken and added 4 mL of 0.1 M DPPH-ethanol solution, reacted in the dark for 30 min. at room temperature. The absorbance was measured at 517 nm (with a 95% ethanol solution as a reference). The calculated DPPH free radical scavenging rates according to the following Formula (1):

(1) $$\mathrm{DPPH}\;\mathrm{free}\;\mathrm{radical}\;\mathrm{scavenging}\;\mathrm{rate}\;\left(\%\right)=\left(1\;-\;\frac{A-A_{i}}{A_{j}}\right)\;\times \;100\%$$

where *A* represented the absorbance of the sample and DPPH-ethanol mixed solution, *A_i_* represented the absorbance of the sample and the 95% ethanol solution, *A_j_* represented the absorbance of DPPH ethanol solution.

#### 2.5.2. ABTS Radical Scavenging Activities

The preparation of ABTS radical cation (ABTS^+^): dissolve 0.0384 g of ABTS solid powder in phosphate buffer solution (0.1 M, pH 7.4), and then diluted to 10 mL with buffer solution to prepare the ABTS solution. Accurately weighed 0.0066 g of potassium persulfate and added to the solution to make the final concentration to 2.45 M, and stored for 16 h in the dark. The ABTS radical cation was diluted with a phosphate buffer solution to make the absorbance at 734 nm of 0.7 ± 0.02.

Sample determination: took 3.0 mL of diluted ABTS radical cation solution, added 30 μL NP, PC, and PCD samples (5 mg/mL), and shaken for 30 s, then avoided light and reacted for 5 min. After reaction, measured the absorbance at 734 nm. The phosphate buffer solution was used as a blank control. The calculated ABTS free radical scavenging rates according to the following Formula (2):

(2) $$\mathrm{ABTS}\;\mathrm{free}\;\mathrm{radical}\;\mathrm{scavenging}\;\mathrm{rate}\;\left(\%\right)=\left(\frac{A_{0\;}-A}{A_{0}}\right)\;\times \;100\%$$

where *A*_0_ represented the absorbance of control and *A* represented the absorbance of sample.

#### 2.5.3. Iron Reduction Activities

Take 1 mL of the NP, PC, and PCD samples (5 mg/mL), added 2.5 mL phosphate buffer (0.2 M pH 6.6) solution and 2.5 mL 1% (*w*/*v*) potassium ferricyanide solution. The reaction was carried out in a water bath at 50 °C for 20 min., then 2.5 mL of 10% (*w*/*v*) trichloroacetic acid was added and centrifuged at 5000 rpm for 10 min. when cooled. 2.5 mL of the supernatant was taken, added 2.5 mL of distilled water, and 0.5 mL of 1% (*w*/*v*) ferric chloride solution. Subsequently, the absorbance at 700 nm was measured after shaking and standing still for 10 min.

### 2.6. Preparation of Nanoemulsions

In aqueous phase, based on our previous research, accurately weigh PPI (Peanut protein isolate), PPI-CA, and PPI-CA-Dex (2%, *w*/*v*), SPI (Soy protein isolate), SPI-CA, and SPI-CA-Dex (3%, *w*/*v*), RBPI (Rice Bran protein isolate), RBPI-CA, and RBPI-CA-Dex (2%, *w*/*v*), and WPI (Whey protein isolate), WPI-CA, and WPI-CA-Dex (2%, *w*/*v*) dried powder, dissolved in 10 mM pH 7.0 phosphate buffer solution to make the corresponding concentrations of aqueous solution, stirred at room temperature overnight to ensure complete dissolution of the powder.

The corn oil was added as oil phase and magnetically stirred at room temperature for 10 min. The crude emulsions were prepared by a homogenization at 10,000 rpm for 4 min. (FJ200-SH, Shanghai Sample Model Factory, Shanghai, China). Subsequently, the ultrasonic processor (Ningbo Xinzhi Biotechnology Co., Ltd., Ningbo, China) was used with ultrasonic power at 500 W for 20 min. The reaction temperature was controlled at 25 °C by ice water bath in order to prevent the reaction temperature from being too high during the preparation.

### 2.7. Droplet Size and Zeta-Potential Measurements of Nanoemulsions

The mean droplet diameters (MDD) of the samples were measured using dynamic light scattering (Zetasizer Nano-ZS90, Malvern Instruments, Worcestershire, UK). The refractive indices of the oil phase and the aqueous phase were set to 1.47 and 1.33, respectively. The zeta-potential (ZP) of the nanoemulsions was measured while using an electrophoresis (Zetasizer Nano-ZS90, Malvern Instruments, Worcestershire, UK). The samples were diluted 100-fold with 10 mM phosphate buffer (pH 7.0) to avoid multiple light scattering effects.

### 2.8. Apparent Viscosity Measurement of Nanoemulsions

The apparent viscosity of the nanoemulsion samples was determined via a rheometer (DHR-1, TA Instruments, Worcestershire, UK) at shear rates of 0.01–100 s^−1^. A 40 mm acrylic parallel plate with a geometric gap of 500 μm was slowly placed on the emulsion sample for 30 s and shear rates measurements were applied at room temperature.

### 2.9. The Stability Determination of Nanoemulsions

#### 2.9.1. Storage Stability

The nanoemulsions were stored at 4 °C for four weeks. The physical stability of the nanoemulsions was characterized at time intervals of one week. The physical stability was evaluated by measuring the change of MDD of nanoemulsions during the storage [23].

#### 2.9.2. Oxidative Stability

The measurement of TBARS (product of the reaction of thiobarbituric acid with malondialdehyde) was determined according to the method that was described by Nejadmansouri et al. [24]. The TBARS levels were established according to 1,1,3,3-tetraethoxypropane standard curve, being measured at 532 nm.

#### 2.9.3. Thermal Stability

The nanoemulsion samples were heated in a boiling water bath for 10 min. and 30 min, cooled to room temperature, and then diluted 100 times with 10 mM pH 7.0 phosphate buffer solution to measure the MDD of the samples [25].

#### 2.9.4. Freeze-Thaw Stability

The nanoemulsion samples were stored in a refrigerator at −20 °C for 20 h and then thawed in a water bath at 35 °C for 2 h. The MDD of the samples were measured by diluting 100 times with 10 mM pH 7.0 phosphate buffer solution [26].

### 2.10. Statistical Analysis

All of the experiments were conducted in triplicate. The results were expressed as mean ± standard deviation. Significance of difference between the means was identified through the Duncan’s multiple-range tests (*p* < 0.05) with SPSS V20.0 (IBM Inc., New York, USA, 2004). Origin V9.1 (OriginLab Inc, Massachusetts, USA, 2013), PeakFit V4.12 (Systat Software Inc., California, USA, 2007) and other software were used for data processing, spectrum analysis, and chart making.

## 3. Results

### 3.1. Structural Analysis of PC and PCD Conjugates

#### 3.1.1. SDS-PAGE Analysis

**Figure 1:**
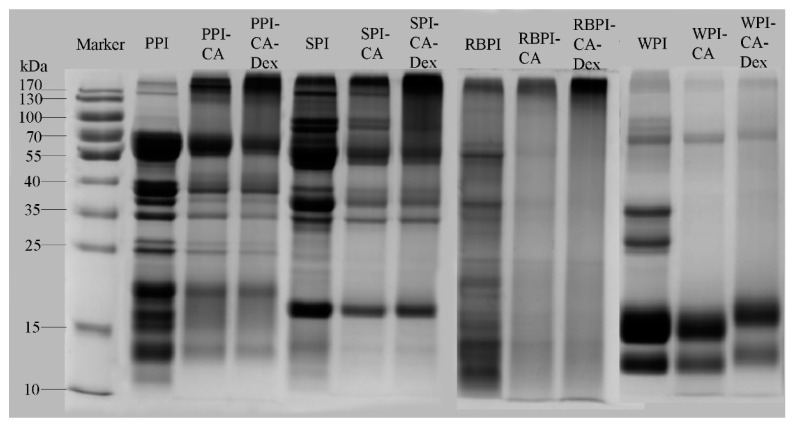
The Sodium Dodecyl Sulfate-Polyacrylamide Gel Electrophoresis (SDS-PAGE) gel electrophoresis spectrums of Native Protein (NP), protein-chlorogenic (PC) and protein-chlorogenic acid-dextran (PCD) samples.

SDS-PAGE is an effective method for analyzing the molecular weight of proteins. The resistance was lower, and the migration speed was faster when small molecular weight protein passed through the gel pore, whereas the tendency of large molecular weight protein was opposite. Figure 1 shows the SDS-PAGE gel electrophoresis spectrums of NP, PC, and PCD samples. As can be seen, low molecular weight electrophoresis bands of PC conjugates were lighter, and the protein migration rates became slower when compared with each NP samples, which indicated that the interaction of protein with CA increased its molecular weight. This experimental result was similar to the electrophoretic results of ovotransferrin-catechin conjugates and whey protein-epigallocatechin gallate (EGCG) conjugates [27]. The band of the PCD conjugates further migrated to the large molecular weight regions, which appeared at the upper end of the gel than PC conjugates. This indicated that dextran interacted with the PC conjugates to form higher molecular weight conjugates, which was consistent with the results of electrophoretic analysis of lactoferrin-polyphenol-polysaccharide conjugates investigated by Liu et al. [10].

#### 3.1.2. Fourier Transform Infrared (FT-IR) Analysis

FT-IR could reflect structural changes in proteins at the level of secondary structure and hydrogen bonding. The FTIR images of NP, PC and PCD were shown in Figure S1. Table 1 showed the contents of the secondary structure of the PC conjugates. As can be seen, when compared to NP, the random coiled structures were significantly increased, and the α-helix structure was significantly decreased. Previous studies [28] have shown that the phenolic grafting reaction resulted in an increase of random coiled structure with a decrease of α-helix structure of the proteins. These changes were due to covalent interactions between proteins and polyphenols, which were consistent with our experimental results.

Subsequently, the grafting with dextran continued to reduce the α-helix content of PCD conjugates, while the percentage of random coiled structure further increased when compared to PC conjugates. This might be due to the uncoiling of the protein under the addition of dextran, induced by the combination of dextran and ε-amino group in protein α-helix region [29]. Mu et al. [30] reported a decrease in α-helix content and an increase in random coil content after the soy protein combined with gum arabic (polysaccharide), which was consistent with our results.

The results of FT-IR showed that the degree of influence of chlorogenic acid and dextran on protein secondary structure vary, depending on protein types. It was observed from Table 1 that the addition of dextran showed the most conspicuous increase in the random coil content occurred in PPI group, while the most significant decrease in the α-helix content appeared in the RBPI group. Through the modification with polyphenols and polysaccharides, protein molecules were unfolded and rearranged, protein structures were changed from order to disorder. Studies have shown that there could be better flexibility for protein molecules [29]. Subsequently, the better corresponding emulsifying properties might be expected, because the higher contents of disordered structures could be more conducive to the protein adsorption at the oil-water interface [30].

#### 3.1.3. Fluorescence Spectroscopy Analysis

**Figure 2:**
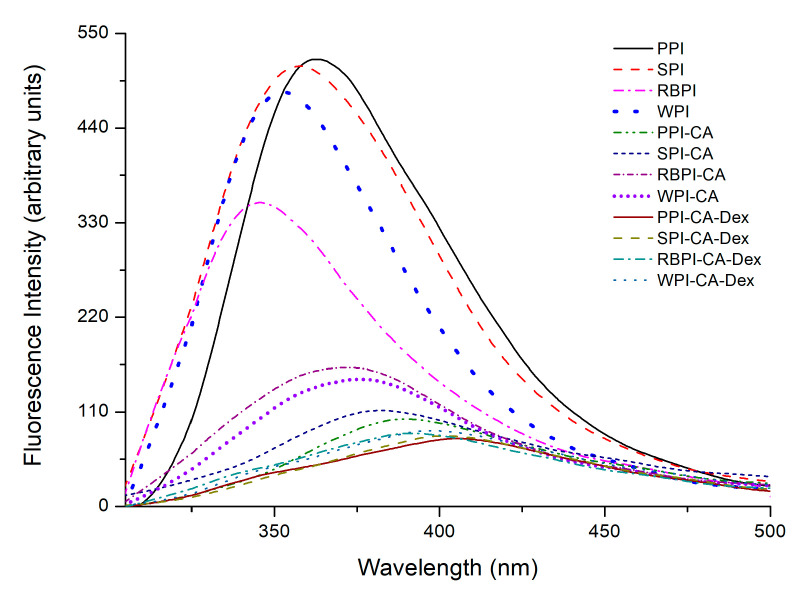
Fluorescence spectra of NP, PC, and PCD samples.

Most proteins could emit inherent fluorescence intensity (% FI) after absorbing ultraviolet light due to the existence of certain amino acid residues in the protein structure (such as Trp, Tyr and Phe) [9]. Previous experimental results showed that the covalent attachment of proteins and polyphenols could cause conformational changes in proteins, which, in turn, led to fluorescence quenching [31]. The fluorescence intensities of the conjugates PPI-CA, SPI-CA, WPI-CA, and RBPI-CA were declined to 80.41%, 78.15%, 54.25%, and 69.26%, respectively, as can be seen from Figure 2. This was probably ascribed to the lower quantum yields induced by hydrophilic polyphenol, leading to the fluorescence quenching of tryptophan via the polyphenol attachment to the protein molecules [32]. Moreover, the fluorescence spectra of PC samples showed slight red shifts after the addition of CA, as seen from Figure 2. The addition of polyphenols caused a certain degree of protein unfolding, as shown in the FT-IR results. The amino acid residues were moved to the protein surfaces and faced a more polar environment [33].

The fluorescence intensities of the PCD conjugates were further reduced when compared to the PC conjugates. In fact, this phenomenon was probably due to the shielding effect of the dextran addition [34]. The maximum emission wavelength (*λ_max_*) of PCD samples exhibited red shift as compared to respective PC samples. It indicated that the addition of dextran changed the micro-environment of tryptophan in the protein, making it more polar to the water phase [35]. Therefore, the protein had a better hydrophilic-hydrophobic balance, which might improve the protein emulsifying properties [36]. Among the four protein samples, the PPI-CA-Dex conjugates showed the most drastic decrease in fluorescence intensity, which could infer that the degree of unfolding and fluorescence quenching of PPI group was the highest. Accordingly, the structure of PPI might change the most, so that it could be predicted that the functional properties were also influenced most.

### 3.2. Properties Analysis of PC and PCD Conjugates

#### 3.2.1. Interfacial Tensions

**Figure 3:**
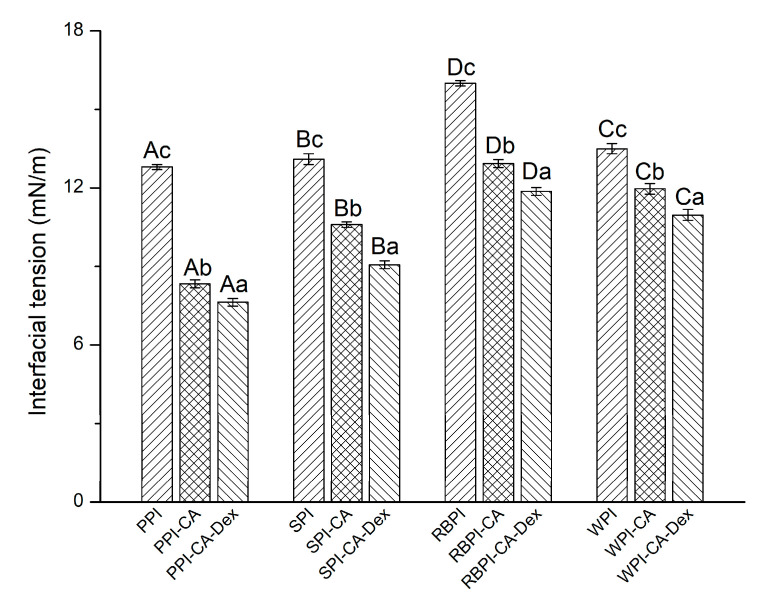
The interfacial tensions of NP, PC, and PCD samples. A–D represented that there were statistically differences (*p* < 0.05) in interfacial tensions among nanoemulsions stabilized by four NPs, four PCs, or four PCDS, a–c represented that there were statistically differences (*p* < 0.05) among NP, PC, and PCD in same proteins.

There has been correlation between interfacial layer rheology of protein and nanoemulsion formation and stability [37]. The measurement of protein interfacial tensions can help us to understand the difference of the four kinds of protein conjugates in forming emulsions. Figure 3 shows the interfacial tensions of the NP, PC, and PCD conjugates. When compared with NP, the interfacial tensions of the PC were significantly reduced (*p* < 0.05). This revealed that the surface activity of the protein was enhanced through grafting with chlorogenic acid. Polyphenol modification could change the structure of protein, as analyzed in the FT-IR results. Protein molecules were de-aggregated, and the structures were further expanded. The connection of hydrophilic groups could reduce the interfacial tension and, thus, formed a more stable nanoemulsion.

It could be seen from Figure 3 that the interfacial tension of the PCD conjugates were significantly lower than each PC conjugates (*p* < 0.05). Among four PCD conjugates, PPI-CA-Dex had the smallest interfacial tension (7.63 ± 0.15 mN/m), while the RBPI-CA-Dex had the largest interfacial tension (11.87 ± 0.15 mN/m). In fact, after the addition of dextran, the dextran could further crosslink with the PC conjugates, leading to the partial unfolding of the protein at the adsorption layer. Thus, dextran induced further unfolding of the protein, as analyzed in FT-IR result, allowing for it to be more efficiently adsorbed at the oil-water interface. Subsequently, thicker viscoelastic membranes were formed with the reduction of the interfacial tension [38].

#### 3.2.2. Analysis of Antioxidant Activities

Table 2 shows the antioxidant activities of the NP, PC, and PCD conjugates. As for PC samples, the antioxidant activity was significantly increased based upon the addition of polyphenol. The covalent binding of protein and polyphenols could cause the deprotonation of the phenolic hydroxyl group and form quinones, which may then react with other chlorogenic acid molecules to form dimerization or other higher polymers [39]. Thus, the free radical scavenging ability and metal ion binding ability of protein could be promoted [40]. Researchers on β-lactoglobulin-catechin conjugate, oval transferrin-catechin conjugate, and lactoferrin-polyphenol conjugate had all obtained similar experimental results as ours [41]. The results of these studies indicated that protein and polyphenols could be combined to obtain antioxidants with fine antioxidant activity.

In addition, it could also be seen from the Table 2 that the antioxidant activities of the PCD conjugates, including DPPH free radical scavenging activities, ABTS free radical scavenging activities, and the iron reducing ability, were significantly higher than PC conjugates (*p* < 0.05). The results showed that PCD conjugates had the best effects on improving the antioxidant activity of the protein. Previous research indicated that the Maillard reaction was an effective method for improving the antioxidant properties, including free radical scavenging, iron chelating activity, and iron reducing ability [42]. On the one hand, the Maillard reaction intermediate compound reductones could act as donators of hydrogen atoms and showed a good ability to break radical chains, thereby improving the DPPH and ABTS free radical scavenging ability of proteins [43]. On the other hand, the improvement of iron reducing ability was mainly due to the formation of high molecular weight polymerization products in the advanced stage of Maillard reactions, which played an important role in improving the iron reducibility. We believed that polyphenols and polysaccharides composite modification produced superimposed and synergistic effects, which further enhanced the antioxidant of the protein.

From the above discussion of the structure and properties of the protein, it could be seen that the polyphenol and polysaccharide modification of the protein could both make the protein secondary structure more disordered and the protein molecules further expanded. Thus, the emulsification properties were speculated to be better, which was further supported by the reduced interfacial tension. In addition, the antioxidant activity of the protein was also advanced with the bonding of polyphenol and polysaccharide. Therefore, further research on modified proteins used as emulsifiers to stabilize nanoemulsions owning high antioxidant ability attracted our interests.

### 3.3. Characterization of PC and PCD Stabilized Nanoemulsion

#### 3.3.1. Mean Droplet Diameters and Zeta-Potential

**Figure 4:**
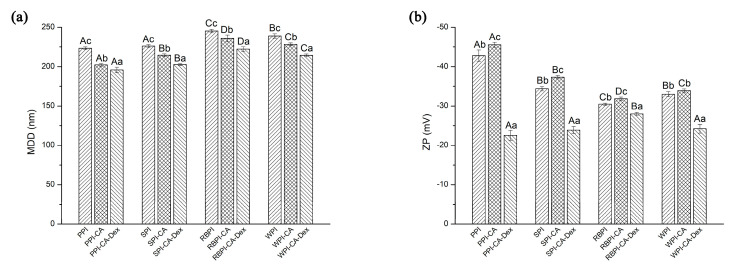
(**a**) and (**b**) showed the mean droplet diameters (MDD) and Zeta-potential (ZP) of the nanoemulsions stabilized by NP, PC, and PCD samples, respectively. A–D represented that there were statistically differences (*p* < 0.05) in MDD or ZP among nanoemulsions stabilized by four NPs, four PCs, or four PCDS, a–c represented that there were statistically differences (*p* < 0.05) among NP, PC, and PCD in same proteins.

Figure 4a,b show the mean droplet diameters (MDD) and Zeta-potential (ZP) of the nanoemulsions. In addition, size distribution by volume and polydispersity index (PDI) of nanoemulsions were given, as shown in Figure S2 and Table S1. When compared with each NP samples, the nanoemulsion stabilized by the PC conjugates significantly decreased in MDD. The PC conjugates effectively inhibited the aggregation of droplets. The addition of polyphenols could increase the surface activity of the protein, which was more conducive to the fragmentation of the droplets, as analyzed in interfacial tensions results. Moreover, the negative charge on the chlorogenic acid group caused the increase of electrostatic repulsion between the oil droplets (Figure 4b), eventually making the droplet size smaller. From Figure 4a, it could be found that the nanoemulsions that were stabilized by PPI-CA-Dex, SPI-CA-Dex, RBPI-CA-Dex, and WPI-CA-Dex conjugates were 195.93 ± 3.40 nm, 202.77 ± 1.12 nm, 222.30 ± 2.90 nm, and 214.30 ± 1.78 nm decreased by 3.13%, 5.47%, 5.73%, and 6.09%, respectively, as compared with each PC conjugates. Nanoemulsion droplets that were stabilized by PCD conjugates became more distributed and more uniform as compared by NP and PC conjugate nanoemulsions. This result indicated that, during the preparation of the nanoemulsions, the PCD conjugates could more effectively suppress the aggregation of droplets. On the one hand, the addition of dextran increased the surface activity of the PCD conjugates, leading to a further reduction of interfacial tension, as shown in Figure 3. This increased protein adsorption effect at the oil-water interface enhanced the spatial repulsion between droplets. On the other hand, as the hydrophilic polysaccharide was adsorbed on the surface of the protein, the hydrophilic region became bulkier. This phenomenon led to a decrease in the filling parameters and an increase in curvature, which ultimately made a decrease in the particle size of nanoemulsions [44].

The ZP of the nanoemulsions stabilized by PPI-CA, SPI-CA, RBPI-CA, and WPI-CA changed from −42.77 to −45.53 mV, −34.40 to −37.30 mV, −30.50 to −31.87 mV, and −33.00 to −33.93 mV, respectively, as can be seen in Figure 4b. The results indicated that the addition of chlorogenic acid increased the amount of surface charge of protein at the interface layer [45]. The absolute value of the ZP for the PC stabilized nanoemulsion increased, which suggested that the electrostatic repulsion between the emulsion droplets was enhanced, which could prevent the droplets from flocculating and improve the stability of the nanoemulsion [45]. The ZP of PPI-CA-Dex, SPI-CA-Dex, RBPI-CA-Dex, and WPI-CA-Dex conjugates stabilized nanoemulsions significantly decreased 50.44%, 35.92%, 12.24%, and 28.59% as compared to nanoemulsions that were stabilized by each PC conjugates (*p* < 0.05). After the modification of the polysaccharide, the absolute values of ZP decreased; this was not beneficial to the dispersion of the droplets. However, large molecular weight polysaccharides increased the steric hindrance of the protein, so it could also effectively prevent the aggregation and flocculation of the droplets. Zhao obtained similar results in the formation of WPI-Dex conjugates’ nanoemulsion [46].

#### 3.3.2. Apparent Viscosity of Nanoemulsion

**Figure 5:**
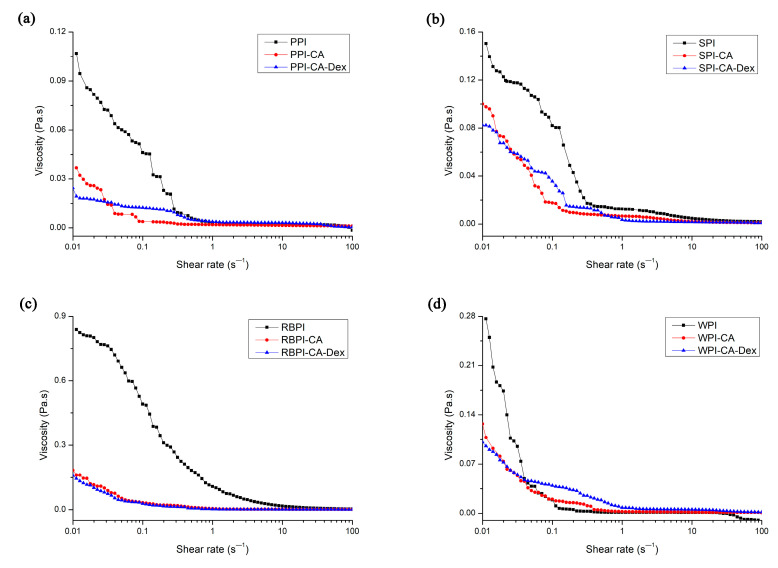
The apparent viscosity of nanoemulsions stabilized by NP, PC, and PCD samples. (**a**) represented the apparent viscosity of nanoemulsions stabilized by PPI, PPI-CA and PPI-CA-Dex; (**b**) represented the apparent viscosity of nanoemulsions stabilized by SPI, SPI-CA and SPI-CA-Dex; (**c**) represented the apparent viscosity of nanoemulsions stabilized by RBPI, RBPI-CA and RBPI-CA-Dex and (**d**) represented the apparent viscosity of nanoemulsions stabilized by WPI, WPI-CA and WPI-CA-Dex.

Rheological behavior was one of the most important properties in food emulsions. Emulsion products with good texture and sensory often required careful control of emulsion rheology [11]. Figure 5 shows the apparent viscosity of NP, PC, and PCD conjugates stabilized nanoemulsions. At low shear rates, all of the PC conjugates stabilized nanoemulsions exhibited shear thinning behavior. After that, the viscosity did not change with an increasing shear rate. The enhanced surface activity of the protein-chlorogenic acid conjugate caused the smaller particle size of the stable nanoemulsion droplets, as analyzed in mean droplet diameters and zeta-potential of nanoemulsions. The smaller particle size made the flow resistance smaller and more orderly in the flow field, thus showing lower apparent viscosity [47].

Four PCD conjugates stabilized nanoemulsions had lower viscosity than respective PC conjugates nanoemulsions. This might be due to the decrease in the interfacial tension of the PCD conjugates after the addition of polysaccharides. The MDD of the nanoemulsion droplets stabilized by PCD conjugates became smaller than PC ones, resulting in smaller flow resistance in the flow field, as shown in Figure 4a. Thus, the viscosity of the PCD stabilized nanoemulsions decreased [3].

### 3.4. Stability of PC, PCD Stabilized Nanoemulsion

#### 3.4.1. Storage Stability

**Figure 6:**
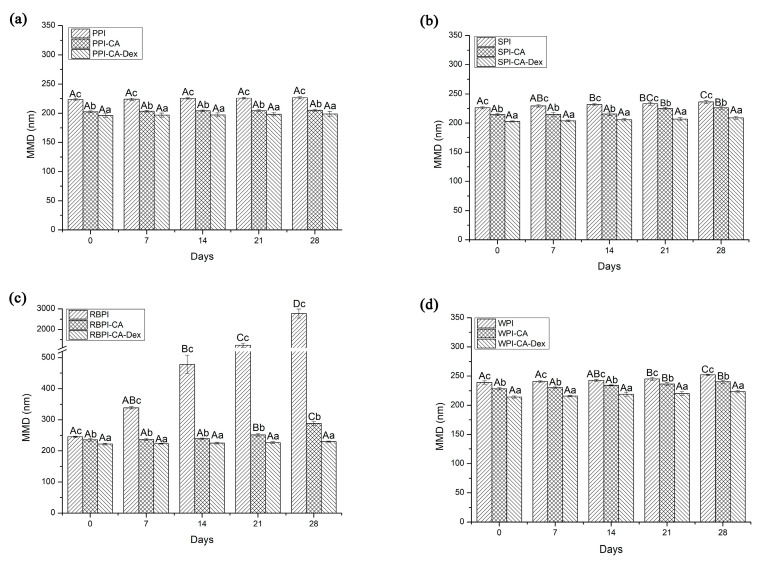
The MDD of nanoemulsions stabilized by NP, PC, and PCD samples under different storage weeks. (**a**) represented the MDD of nanoemulsions stabilized by PPI, PPI-CA and PPI-CA-Dex under different storage weeks; (**b**) represented the MDD of nanoemulsions stabilized by SPI, SPI-CA and SPI-CA-Dex under different storage weeks; (**c**) represented the MDD of nanoemulsions stabilized by RBPI, RBPI-CA and RBPI-CA-Dex under different storage weeks; (**d**) represented the MDD of nanoemulsions stabilized by WPI, WPI-CA and WPI-CA-Dex under different storage weeks. A–D represented that there were statistically differences (*p* < 0.05) in MDD in different storage time of same samples, a–c represented that there were statistically differences (*p* < 0.05) in MDD in same storage time of different samples.

The MDD of nanoemulsions stabilized by SPI and RBPI increased significantly on the second week of storage (*p* < 0.05), as shown in Figure 6. When compared with the NP groups, the storage stability of the PC groups was greatly improved. This might be due to the combination of chlorogenic acid, which increased the zeta-potential of the droplet interface protective layer, and then formed a stable nanoemulsion through stronger electrostatic repulsion. The MDD of nanoemulsions stabilized by SPI-CA and RBPI-CA significantly increased on the third week of storage (*p* < 0.05). However, the MDD changes of PPI and PPI-CA stabilized nanoemulsions were not significant (*p* > 0.05). This showed that PPI itself had strong storage stability as emulsifier. The WPI and WPI-CA stabilized nanoemulsions both increased particle size on the third week of storage, showing that the influence of chlorogenic acid on the stability of WPI-emulsified nanoemulsions was small.

During the entire four-week storage, the MDD of nanoemulsions that were stabilized by four PCD conjugates all kept constant (*p* > 0.05). Nanoemulsions stabilized by PCD conjugates as emulsifiers showed the best storage stability after the storage of four weeks. Firstly, the PCD conjugates had the lowest MDD in the initial stage, as shown in Figure 4a, so that they could more effectively suppress droplet aggregation. Secondly, previous studies have found that the attachment of polysaccharides with a molecular weight ≥5 kDa (the molecular weight of dextran used in this experiment was 40 kDa) could provide steric repulsion of protein emulsifiers films and made nanoemulsions with stability against droplet aggregation [48]. The thicker droplet interfacial membranes were formed after the dextran compounding, so that the steric repulsion effect of the PCD conjugates that adsorbed on the interface was enhanced. Thus, the droplets could be protected from aggregation.

#### 3.4.2. Oxidation Stability

**Figure 7:**
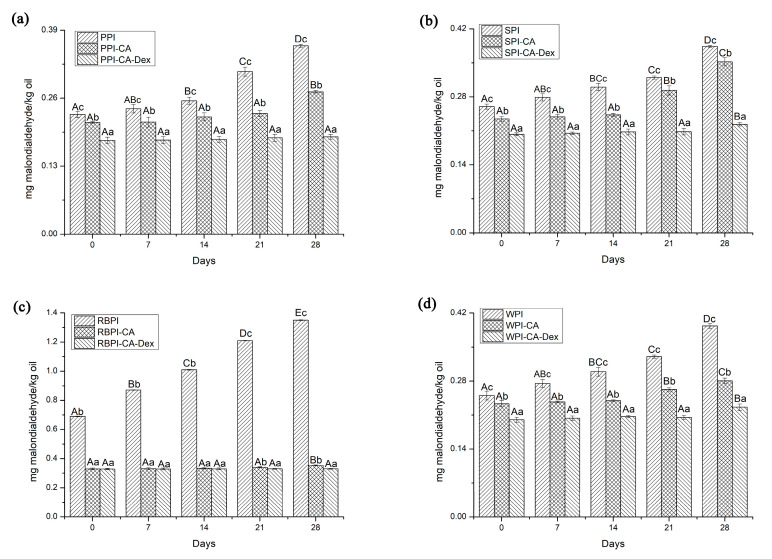
The thiobarbituric acid-reactive substances (TBARS) values of nanoemulsions stabilized by NP, PC, and PCD samples under different storage weeks. (**a**) represented the TBARS values of nanoemulsions stabilized by PPI, PPI-CA and PPI-CA-Dex under different storage weeks; (**b**) represented the TBARS values of nanoemulsions stabilized by SPI, SPI-CA and SPI-CA-Dex under different storage weeks; (**c**) represented the TBARS values of nanoemulsions stabilized by RBPI, RBPI-CA and RBPI-CA-Dex under different storage weeks; (**d**) represented the TBARS values of nanoemulsions stabilized by WPI, WPI-CA and WPI-CA-Dex under different storage weeks. A–D represented that there were statistically differences (*p* < 0.05) in TBARS values in different storage time of same samples, a–c represented that there were statistically differences (*p* < 0.05) in TBARS values in same storage time of different samples.

Studies have shown that protein-polysaccharide or protein-polyphenol conjugates could both effectively inhibit the oxidation of emulsions as interfacial antioxidants. Lipid oxidation secondary product (malonaldehyde) could interact with thiobarbituric acid (TBA) to form a pink compound (TBARS). Its absorbance values can reflect the degree of oxidation of the nanoemulsion and could be detected at 532 nm. Therefore, TBARS was used to evaluate the oxidative stability of the emulsion. It can be seen from Figure 7 that the TBARS values of the NP stabilized nanoemulsions significantly increased each week. The PPI-CA and RBPI-CA stabilized nanoemulsions could remain constant in three weeks, while two weeks was observed for the SPI-CA and WPI-CA groups.

Accordingly, the ability of PCD conjugates stabilized nanoemulsions was most improved. For four weeks storage, the TBARS values of the PCD conjugates stabilized nanoemulsions were all lower than those of the PC conjugates, as seen from Figure 7. The TBARS values of PPI-CA-Dex and RBPI-CA-Dex conjugates stabilized nanoemulsions were not significantly increased throughout the whole storage period (*p* > 0.05), as shown in Figure 7a,c. From Figure 7b,d, compared with each PC, SPI-CA-Dex and WPI-CA-Dex could be prolonged for another week to keep the TBARS values unchanged (*p* > 0.05). This might be because that PCD conjugates could form a dense and thick interfacial layer on the surface of the droplet to act as a space barrier as analysis in interfacial tensions, physically prevented the interaction of pro-oxidants and lipids, which in turn acted to inhibit the oxidation of nanoemulsions [49]. In addition, the antioxidant capacity of PCD conjugates themselves was the other positive factor that should not be ignored for nanoemulsions oxidative stability. Studies have shown that there was a correlation between the number of polyphenols linked to protein and the degree of oxidation [9]. The higher the amount of CA bounded in the protein, the lower degree of oxidation in the nanoemulsion. The fluorescence quenching of PPI was highest, thereby this might be the reason why PPI group had the strongest antioxidant capacity among the four proteins, as analyzed in fluorescence spectroscopy.

#### 3.4.3. Thermal Stability

**Figure 8:**
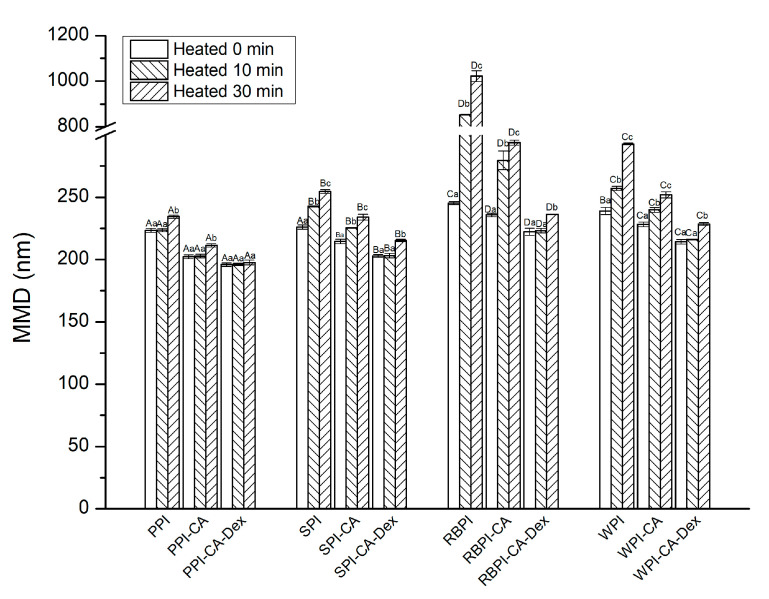
The MDD of nanoemulsions stabilized by NP, PC, and PCD samples after heating treatment. A–D represented that there were statistically differences (*p* < 0.05) in MDD among nanoemulsions stabilized by four NPs, four PCs or four PCDS in same heat conditions; a–c represented that there were statistically differences (*p* < 0.05) in MDD among same samples in different heat conditions.

Figure 8 shows the effect of heating on the stability of the NP, PC, and PCD conjugates stabilized nanoemulsions. When compared with NP stabilized nanoemulsions, PC stabilized nanoemulsions showed significantly smaller MDD after heating treatment (*p* < 0.05). In general, as the heat treatment time was extended, the growth rate of droplet size accelerated and the aggregation between the droplets usually became more intense. The adsorbed protein partially expanded with the extension of the heat treatment time, further leading to the hydrophobic attraction to accelerate droplets aggregation [50]. However, the MDD of PC stabilized nanoemulsions increased relatively slowly, it might be because, as follows: (1) the modification of polyphenols made its structure relatively disordered (Table 1), heat treatment might not fully expand the protein [51]; (2) for PC stabilized nanoemulsions, the particle size was relatively small and the ZP was relatively high (Figure 4a), which might improve the stability of the nanoemulsion under heat treatment.

The MDD of PPI-CA-Dex stabilized nanoemulsions kept steady after heat treatment for 30 min. *p* > 0.05 exhibited good resistance to droplet aggregation. The SPI-CA-Dex, WPI-CA-Dex and RBPI-CA-Dex stabilized nanoemulsions had smaller MDD increase after heat treatment for 30 min. than PC stabilized nanoemulsions. That was mainly because, when the protein grafted with dextran, the thermal denaturation temperature of the protein increased, which led to the improvement of the thermal stability of the PCD conjugates that were stabilized nanoemulsion.

#### 3.4.4. Freeze-Thaw Stability

**Figure 9:**
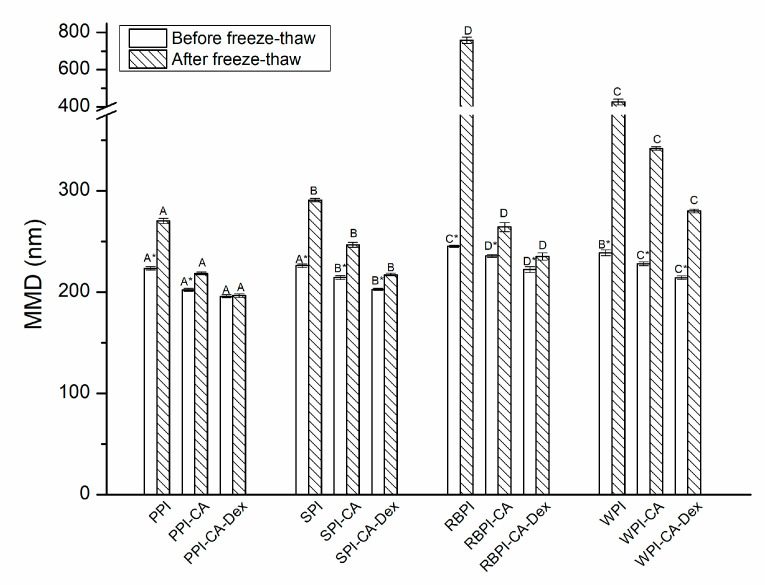
The MDD of nanoemulsions stabilized by NP, PC, and PCD samples after freeze-thaw treatment. A–D represented that there were statistically differences (*p* < 0.05) in MDD among nanoemulsions stabilized by four NPs, four PCs or four PCDS. * represented that there were statistically differences (*p* < 0.05) in MDD between same samples after freeze-thaw treatment.

The MDD of the NP stabilized nanoemulsions changed greatly after the freeze-thaw treatment, as shown in Figure 9. The MDD of the PPI, SPI, and WPI-stabilized nanoemulsion increased by 21.04%, 28.69%, and 78.59%, respectively, while the RBPI-stabilized nanoemulsion increased for 209%. The MDD of the PC conjugate stabilized nanoemulsion increased lower compared with each NP stabilized nanoemulsions, for about 8.01%, 15.01%, 12.14%, and 49.83%. The results showed that PC stabilized nanoemulsions had better freeze-thaw stability when compared with each NP stabilized nanoemulsions. One reason for this improvement, as analyzed in ZP of nanoemulsions, the addition of chlorogenic acid introduced more hydroxyl groups into the protein molecule and changed the interface surface charge, which increases the electrostatic repulsion between droplets.

When compared with PC conjugates stabilized nanoemulsions, PCD conjugates stabilized nanoemulsions had the smallest increase in MDD. The MDD of PPI-CA-Dex, SPI -CA-Dex, WPI-CA-Dex, and RBPI-CA-Dex stabilized nanoemulsion increased by 0.34%, 7.20%, 5.79%, and 30.74%, respectively. Among them, the MDD of PPI-CA-Dex stabilized nanoemulsions did not change significantly after freezing and thawing (*p* > 0.05). The spatial repulsion between droplets of PCD conjugates stabilized nanoemulsions was higher than that of PC ones, which was also the main reason to improve the freeze-thaw stability for PCD conjugates. Furthermore, from NP to PC conjugates to PCD conjugates, the adsorption effect at the interface got better because the interfacial tension became smaller, which gained more resistant to aggregation of the droplets, as can be seen in Figure 3. Therefore, the PCD conjugates stabilized nanoemulsion had the best freeze-thaw stability.

## 4. Conclusions

Herein, for the PCD conjugates, a combination of polyphenols and polysaccharides improved the emulsifying and antioxidant properties of native proteins. When compared with PC conjugates and NPs, the PCD conjugates had larger molecular weight according to the band that appeared at the upper end of the gel (Figure 1). After bonded to chlorogenic acid and dextran, the protein structure became disorder and the amino acid residues inside the protein were exposed to the polar environment. Structure changes caused the interfacial tension reduced and antioxidant activity enhanced in varying degrees. The PCD stabilized nanoemulsions, when compared to the nanoemulsions stabilized by PC conjugates and NPs, showed smallest particle size and range changes during the 28-day storage, which showed best storage stability. What is more, PCD conjugates also had significantly improved oxidation stability, thermal stability, and freeze-thaw stability of nanoemulsions. This work has shown that chlorogenic acid and dextran modification on protein could change the original structure of the protein and improved its functional properties to obtain nanoemulsions with better stability. In summary, PCD conjugates could be considered as the more suitable emulsifiers to prepare nanoemulsions.

## Figures and Tables

**Table 1:** The secondary structure contents of NP, PC, and PCD samples.

Samples	α-Helix (%)	β-Sheet (%)	β-Turn (%)	Random Curl (%)
PPI	12.01 ± 0.03 ^c^	30.17 ± 0.03 ^c^	41.26 ± 0.03 ^a^	16.56 ± 0.03 ^a^
PPI-CA	11.94 ± 0.03 ^b^	29.70 ± 0.02 ^b^	41.52 ± 0.02 ^b^	16.84 ± 0.01 ^b^
PPI-CA-Dex	11.05 ± 0.03 ^a^	28.65 ± 0.01 ^a^	41.94 ± 0.02 ^c^	18.36 ± 0.02 ^c^
SPI	12.84 ± 0.02 ^c^	31.44 ± 0.03 ^c^	38.99 ± 0.04 ^a^	16.73 ± 0.02 ^a^
SPI-CA	12.49 ± 0.02 ^b^	30.89 ± 0.02 ^b^	39.29 ± 0.01 ^b^	17.33 ± 0.01 ^b^
SPI-CA-Dex	11.60 ± 0.01 ^a^	30.71 ± 0.02 ^a^	39.71 ± 0.01 ^c^	17.98 ± 0.01 ^c^
RBPI	16.90 ± 0.01 ^c^	34.63 ± 0.03 ^c^	34.64 ± 0.02 ^a^	13.83 ± 0.02 ^a^
RBPI-CA	15.89 ± 0.02 ^b^	33.30 ± 0.02 ^b^	35.99 ± 0.01 ^b^	14.82 ± 0.02 ^b^
RBPI-CA-Dex	15.21 ± 0.01 ^a^	32.84 ± 0.01 ^a^	36.37 ± 0.01 ^c^	15.58 ± 0.02 ^c^
WPI	15.68 ± 0.02 ^c^	35.65 ± 0.02 ^c^	34.11 ± 0.04 ^a^	14.56 ± 0.03 ^a^
WPI-CA	14.82 ± 0.02 ^b^	35.07 ± 0.02 ^b^	34.68 ± 0.01 ^b^	15.43 ± 0.02 ^b^
WPI-CA-Dex	14.35 ± 0.01 ^a^	34.71 ± 0.02 ^a^	34.77 ± 0.02 ^c^	16.17 ± 0.02 ^c^

a–c indicated the significant difference between NP, PC and PCD samples under the same protein and same structure (*p* < 0.05).

**Table 2:** 1,1-Diphenyl-2-picrylhydrazyl radical 2,2-Diphenyl-1-(2,4,6-trinitrophenyl)hydrazyl (DPPH) free radical scavenging activities, 2,2′-amino-di(2-ethyl-benzothiazoline sulphonic acid-6)ammonium salt (ABTS) free radical scavenging activities, and the iron reducing ability of NP, PC, and PCD samples.

Samples	DPPH Free Radical Scavenging Ability (%)	ABTS Free Radical Scavenging Ability (%)	Iron Reducing Ability (Abs)
PPI	23.6 ± 0.3 ^a^	22.4 ± 0.3 ^a^	0.063 ± 0.002 ^a^
PPI-CA	62.2 ± 0.6 ^b^	95.7 ± 0.3 ^b^	0.340 ± 0.002 ^b^
PPI-CA-Dex	68.1 ± 0.5 ^c^	97.1 ± 0.3 ^c^	0.373 ± 0.002 ^c^
SPI	22.0 ± 0.3 ^a^	21.1 ± 0.2 ^a^	0.029 ± 0.002 ^a^
SPI-CA	55.7 ± 0.5 ^b^	83.4 ± 0.3 ^b^	0.265 ± 0.002 ^b^
SPI-CA-Dex	59.6 ± 0.2 ^c^	86.5 ± 0.4 ^c^	0.295 ± 0.001 ^c^
RBPI	18.7 ± 0.4 ^a^	17.7 ± 0.2 ^a^	0 ^a^
RBPI-CA	57.9 ± 0.4 ^b^	90.0 ± 0.2 ^b^	0.314 ± 0.002 ^b^
RBPI-CA-Dex	62.4 ± 0.3 ^c^	94.3 ± 0.3 ^c^	0.360 ± 0.002 ^c^
WPI	21.7 ± 0.2 ^a^	20.0 ± 0.3 ^a^	0 ^a^
WPI-CA	54.8 ± 0.6 ^b^	77.5 ± 0.4 ^b^	0.264 ± 0.002 ^b^
WPI-CA-Dex	58.6 ± 0.3 ^c^	80.8 ± 0.3 ^c^	0.292 ± 0.002 ^c^

a–c indicated the significant difference between NP, PC and PCD samples under the same protein and same antioxidant activity (*p* < 0.05)

## Supplementary Materials

The following are available online at https://www.mdpi.com/2079-4991/10/6/1094/s1, Figure S1: FTIR images of NP, PC and PCD. Figure S2: Size distribution by volume of nanoemulsions stabilized by NP, PC and PCD. Table S1: The polydispersity index (PDI) of nanoemulsions stabilized by NP, PC and PCD.

## Abbreviations

ABTS	2,2′-amino-di(2-ethyl-benzothiazoline sulphonic acid-6)ammonium salt
BHT	butylated hydroxytoluene
DPPH	1,1-Diphenyl-2-picrylhydrazyl radical 2,2-Diphenyl-1-(2,4,6-trinitrophenyl)hydrazyl
EGCG	epigallocatechin gallate
FTIR	Fourier Transform Infrared
IT	Interfacial Tensions
MDD	mean droplet diameters
PC	protein-chlorogenic acid
PCD	protein-chlorogenic acid-dextran
PPI	peanut protein isolate
RBPI	rice bran protein isolate
SDS-PAGE	Sodium Dodecyl Sulfate-Polyacrylamide Gel Electrophoresis
SPI	soybean protein isolate
WPI	whey protein isolate
