# Supplementary material for: The Improvement of Nanoemulsion Stability and Antioxidation via Protein-Chlorogenic Acid-Dextran Conjugates as Emulsifiers
Source: Nanomaterials (Basel). 2020 Jun 1;10(6):1094. doi: 10.3390/nano10061094 (PMC7353371; doi:10.3390/nano10061094)
Supplement: Supplementary file 1 [file nanomaterials-10-01094-s001.pdf]

nanomaterials

Supplementary Material

The Improvement of Nanoemulsion Stability and Antioxidation via Protein-Chlorogenic Acid-Dextran Conjugates as Emulsifiers

Chang Liu [1,†], Hua Jin [1,†], Yue Yu [1], Jingying Sun [1], Huanyu Zheng [2,3,4], Yan Zhang [5], Jing Xu [1,*], and Xiuqing Zhu [6,*]

[1] College of Art and Science, Northeast Agricultural University, Harbin 150030, Heilongjiang, China; lovekursaal@163.com (C.L.); jinhua@neau.edu.cn (H.J.); Y13206689606@163.com (Y.Y.); asunjinying@126.com (J.S.)

[2] College of Food Science, Northeast Agricultural University, Harbin,150030, China; zhenghuanyu1@163.com

[3] Heilongjiang Green Food Science Research Institute, Harbin 150028, Heilongjiang, China

[4] National Research Center of Soybean Engineering and Technology, Harbin 150028, Heilongjiang, China

[5] Coastal Research and Extension Center, Mississippi State University, MS 39762, United States; yzhang@fsnhp.msstate.edu

[6] College of Food Engineering, Harbin University of Commerce; Key Laboratory of Grain Food and Comprehensive Processing of Grain Resource of Heilongjiang Province, Harbin 150076, Heilongjiang, China

* Correspondence:xujing@neau.edu.cn (J.X.); xqzhuwang@163.com (X.Z.); Tel.: +86-1379-665-2155 (J.X.); +86-1384-510-7825 (X.Z.)

† These authors contributed equally to this work and should be considered co-first authors.

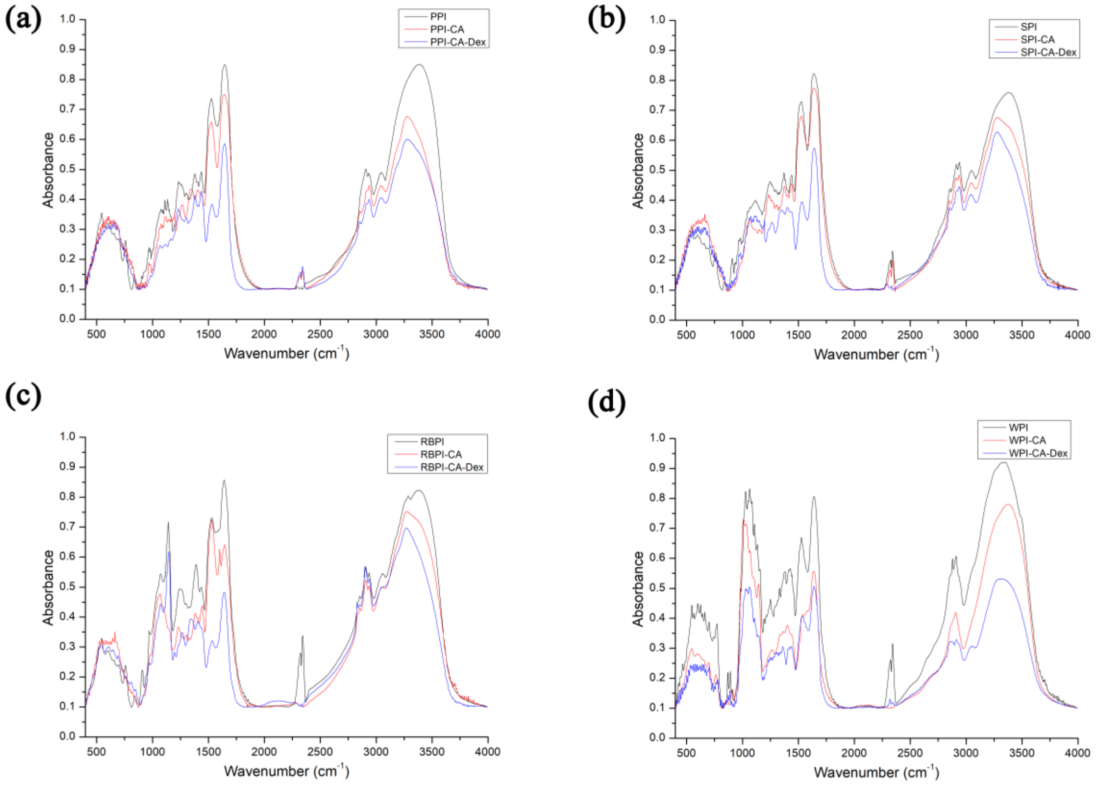

Figure S1. FTIR images of NP, PC and PCD. (**a**) represented FTIR images of PPI, PPI-CA and PPI-CA-Dex, respectively; (**b**) FTIR images of SPI, SPI-CA and SPI-CA-Dex, respectively; (**c**) FTIR images of RBPI, RBPI-CA and RBPI-CA-Dex, respectively; (**d**) represented FTIR images of WPI, WPI-CA and WPI-CA-Dex, respectively.

(1a)

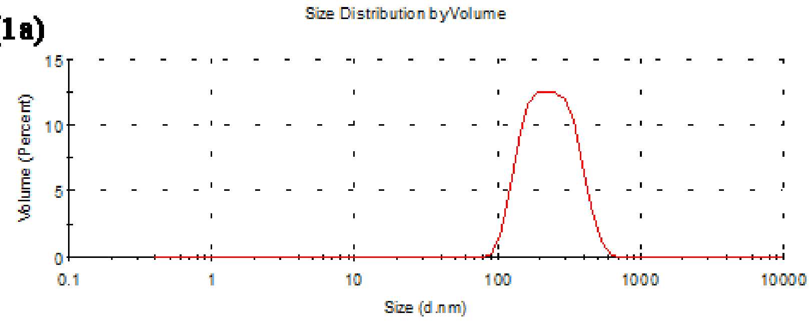
Size Distribution by Volume
Volume (Percent)
15
10
5
0
0.1
1
10
100
1000
10000
Size (d.nm)

(1b)

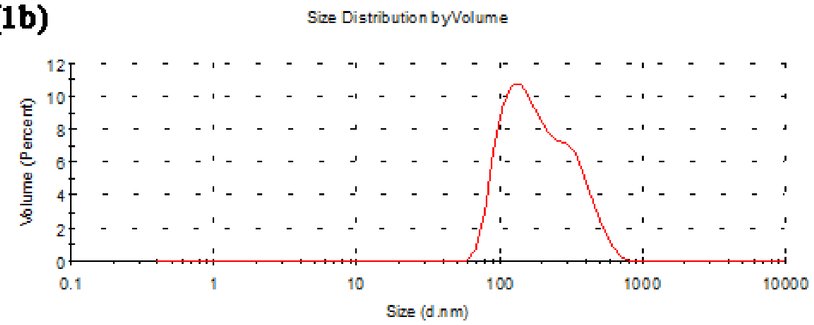
Size Distribution by Volume
Volume (Percent)
12
10
8
6
4
2
0
0.1
1
10
100
1000
10000
Size (d.nm)

(1c)

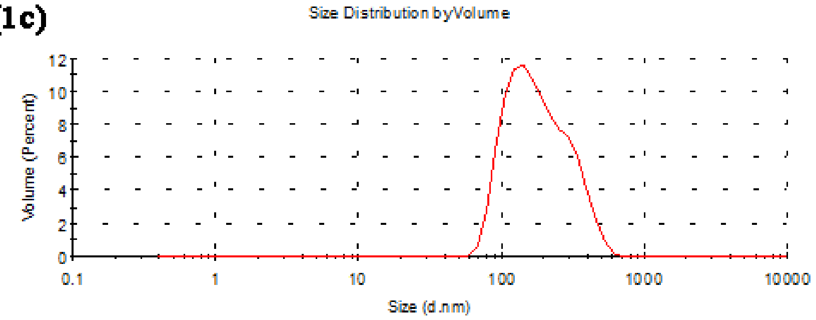
Size Distribution by Volume
Volume (Percent)
12
10
8
6
4
2
0
0.1
1
10
100
1000
10000
Size (d.nm)

(2a)

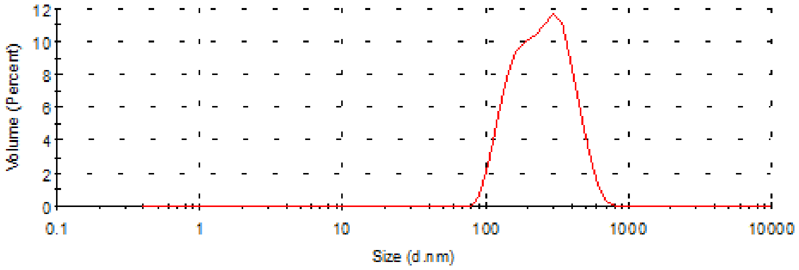
Size Distribution byVolume
Volume (Percent)
0
2
4
6
8
10
12
0.1
1
10
100
1000
10000
Size (d.nm)

(2b)

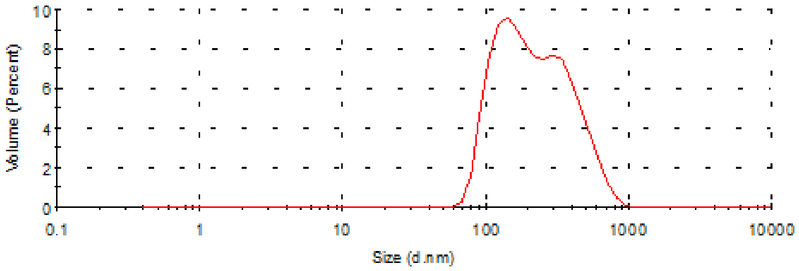
Size Distribution byVolume
Volume (Percent)
0
2
4
6
8
10
0.1
1
10
100
1000
10000
Size (d.nm)

(2c)

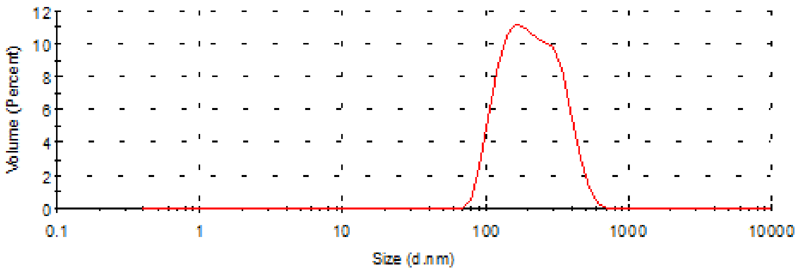
Size Distribution byVolume
Volume (Percent)
0
2
4
6
8
10
12
0.1
1
10
100
1000
10000
Size (d.nm)

(3a)

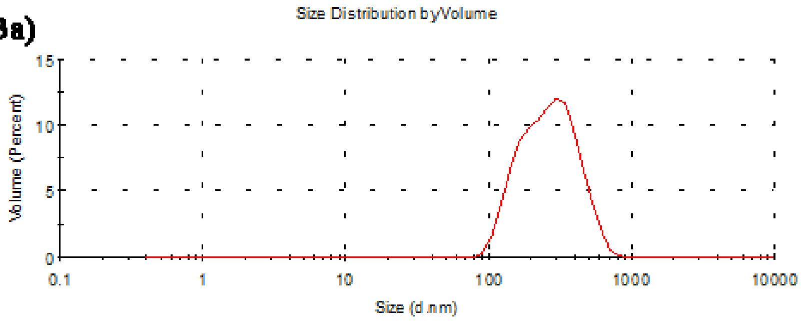
Size Distribution byVolume
Volume (Percent)
15
10
5
0
0.1
1
10
100
1000
10000
Size (d.nm)

(3b)

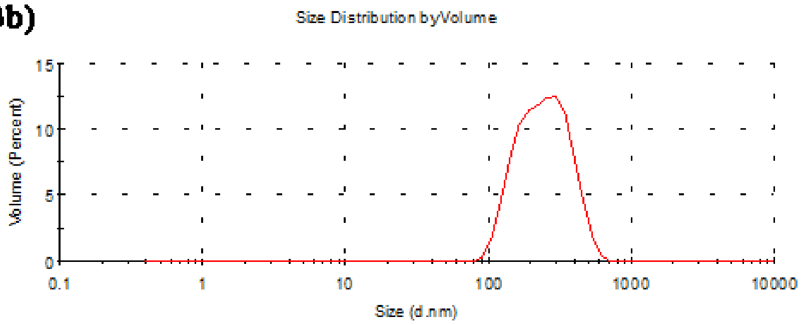
Size Distribution byVolume
Volume (Percent)
15
10
5
0
0.1
1
10
100
1000
10000
Size (d.nm)

(3c)

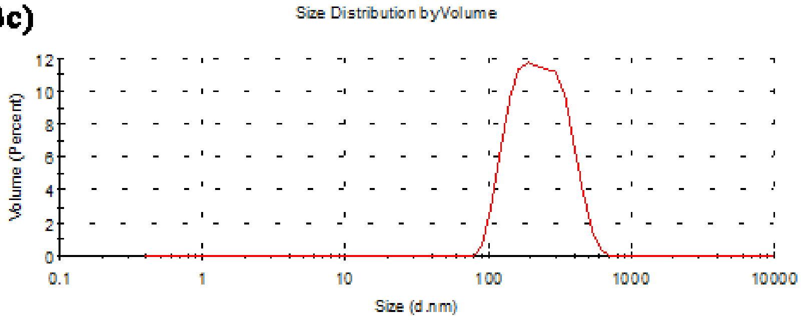
Size Distribution byVolume
Volume (Percent)
12
10
8
6
4
2
0
0.1
1
10
100
1000
10000
Size (d.nm)

(4a)

Size Distribution byVolume

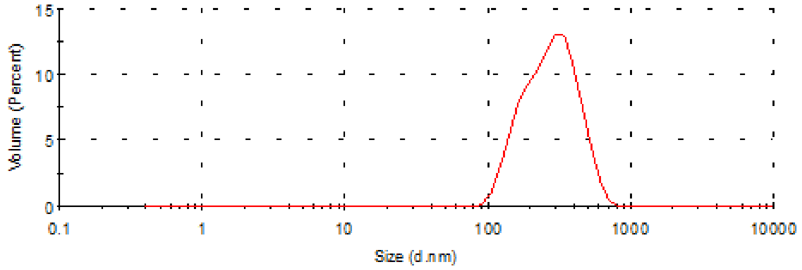

(4b)

Size Distribution byVolume

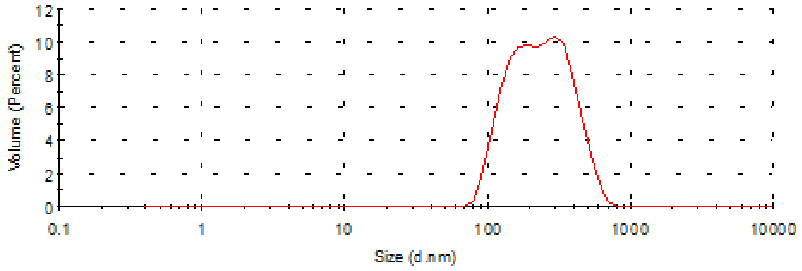

(4c)

Size Distribution byVolume

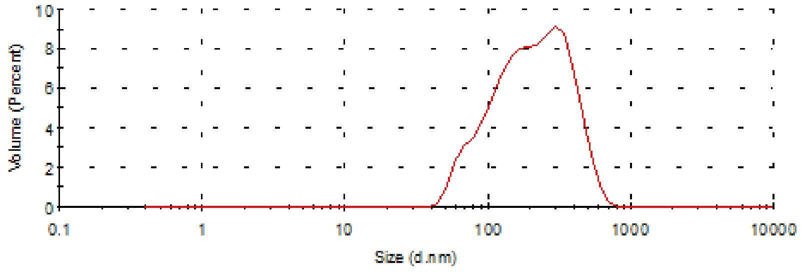

Figure S2. Size distribution by volume of nanoemulsions stabilized by NP, PC and PCD. (**1a**–**1c**) represented the size distribution of nanoemulsions stabilized by PPI, PPI-CA and PPI-CA-Dex, respectively; (**2a**–**2c**) represented the size distribution of nanoemulsions stabilized by SPI, SPI-CA and SPI-CA-Dex, respectively; (**3a**–**3c**) represented the size distribution of nanoemulsions stabilized by RBPI, RBPI-CA and RBPI-CA-Dex, respectively; (**4a**–**4c**) represented the size distribution of nanoemulsions stabilized by WPI, WPI-CA and WPI-CA-Dex, respectively.

Table S1. The polydispersity index (PDI) of nanoemulsions stabilized by NP, PC and PCD.

Nanoemulsions stabilized by	PDI
PPI	0.090
PPI-CA	0.177
PPI-CA-Dex	0.150
SPI	0.115
SPI-CA	0.169
SPI-CA-Dex	0.142
RBPI	0.168
RBPI-CA	0.099
RBPI-CA-Dex	0.144
WPI	0.160
WPI-CA	0.166
WPI-CA-Dex	0.138
